# Circulating KCNH2 Current-Activating Factor in Patients with Heart Failure and Ventricular Tachyarrhythmia

**DOI:** 10.1371/journal.pone.0019897

**Published:** 2011-05-19

**Authors:** Hiroki Sugiyama, Kazufumi Nakamura, Hiroshi Morita, Satoshi Akagi, Yoshinori Tani, Yusuke Katayama, Nobuhiro Nishii, Toru Miyoshi, Satoshi Nagase, Kunihisa Kohno, Kengo Fukushima Kusano, Tohru Ohe, Junko Kurokawa, Tetsushi Furukawa, Hiroshi Ito

**Affiliations:** 1 Department of Cardiovascular Medicine, Okayama University Graduate School of Medicine, Dentistry and Pharmaceutical Sciences, Okayama, Japan; 2 Department of Cardiovascular Therapeutics, Okayama University Graduate School of Medicine, Dentistry and Pharmaceutical Sciences, Okayama, Japan; 3 Department of Bio-Informational Pharmacology, Medical Research Institute, Tokyo Medical and Dental University, Tokyo, Japan; Heart Center Munich, Germany

## Abstract

**Background:**

It is estimated that approximately half of the deaths in patients with HF are sudden and that the most likely causes of sudden death are lethal ventricular tachyarrhythmias such as ventricular tachycardia (VT) or fibrillation (VF). However, the precise mechanism of ventricular tachyarrhythmias remains unknown. The KCNH2 channel conducting the delayed rectifier K^+^ current (I_Kr_) is recognized as the most susceptible channel in acquired long QT syndrome. Recent findings have revealed that not only suppression but also enhancement of I_Kr_ increase vulnerability to major arrhythmic events, as seen in short QT syndrome. Therefore, we investigated the existence of a circulating KCNH2 current-modifying factor in patients with HF.

**Methodology/Principal Findings:**

We examined the effects of serum of HF patients on recombinant I_Kr_ recorded from HEK 293 cells stably expressing KCNH2 by using the whole-cell patch-clamp technique. Study subjects were 14 patients with non-ischemic HF and 6 normal controls. Seven patients had a history of documented ventricular tachyarrhythmias (VT: 7 and VF: 1). Overnight treatment with 2% serum obtained from HF patients with ventricular arrhythmia resulted in a significant enhancement in the peaks of I_Kr_ tail currents compared to the serum from normal controls and HF patients without ventricular arrhythmia.

**Conclusions/Significance:**

Here we provide the first evidence for the presence of a circulating KCNH2 channel activator in patients with HF and ventricular tachyarrhythmias. This factor may be responsible for arhythmogenesis in patients with HF.

## Introduction

Heart failure (HF) remains a major clinical problem all over the world.[1] It is estimated that approximately half of the deaths in these patients is sudden and that the most likely causes of sudden cardiac death (SCD) are lethal cardiac arrhythmia such as ventricular tachycardia (VT) or fibrillation (VF).[2] It has been known for a long time that the failing heart undergoes a complex electrical remodeling of ventricular myocytes and that a consequent reduction of repolarization reserve and electrical instability may predispose to an increased risk of life-threatening arrhythmia.[3], [4]

KCNH2, also called human ether-a-go-go-related gene (hERG) potassium channel, is responsible for the rapid components of delayed rectifier potassium currents (I_Kr_). I_Kr_ is a major contributor to the repolarization process of cardiac action potentials and is recognized as the most susceptible channel in acquired long QT syndrome.[5] Recent findings have revealed that not only suppression but also enhancement of I_Kr_ increase vulnerability to major arrhythmic events. A gain-of-function mutation in KCNH2 has also gained recognition as a congenital disorder characterized by a higher risk for major arrhythmic events and SCD, referred to as short QT syndrome (SQT).[6], [7] It has also been reported that PD-118057, an I_Kr_-activating agent, predisposes to cardiac arrhythmias *in vitro*.[8] HF also induces remodeling of I_Kr_, but there is no widespread consensus on suppression or enhancement of I_Kr_ in previous studies.[9], [10] Furthermore, despite the incremental progress in understanding intrinsic I_Kr_ modulators, to our knowledge, there has been no report of naturally occurring substances with a KCNH2 current-activating effect in patients with HF. Therefore, we investigated the existence of a circulating KCNH2 current-activating factor in patients with HF.

## Results

### Characteristics of the study subjects

Demographics and selected clinical characteristics of the HF patients are summarized in Table 1. The etiology of HF in the study population consisted of DCM (n = 8), documented myocoarditis (n = 3), dilated hypertrophic cardiomyopathy (n = 1), tachycardia-induced cardiomyopathy (n = 1) and apical ballooning syndrome (n = 1). Ventricular tachyarrhythmias were documented in 8 patients: non-sustained VT in 4 (50%), sustained VT in 3 (38%) and VF in 1 (13%). There were no significant differences in the mean age between the control group, the HF without VT and VF (VT/VF (−)) group, and the HF with VT and VF (VT/VF (+)) group (42.7±14.8 yrs, 50.7±10.9 yrs and 53.3±10.8 yrs, respectively; *P* = 0.28). Male-to-female ratio was also not significantly different between the groups (2∶1, 2∶1 and 1∶1 for the control, VT/VF (−) and VT/VF (+) groups, respectively; *P* = 0.76). Mean QTc interval was longer in the overall HF patients than in the controls (0.46±0.03 sec^1/2^ vs. 0.42 ± 0.03 sec^1/2^; *P*<0.05), but there was no significant difference between the VT/VF (−) and VT/VF (+) groups (0.45±0.03 sec^1/2^ vs. 0.47±0.02 sec^1/2^; *P* = 0.51). Mean left ventricular ejection fraction (EF) was similar in the VT/VF (−) and VT/VF (+) groups (39±11% vs. 31±13%, *P* = 0.22).

**Table 1:** Clinical characteristics of the study population.

Case No.	Age/Sex	Diagnosis	NYHA	LVEF (%)	Arrhythmia	QTc (sec^1/2^)
1	73/F	myocarditis	II	50	sustained VT	0.46
2	45/M	DCM	II	11	NSVT	0.47
3	55/F	dilated-HCM	IV	25	NSVT	0.47
4	37/F	DCM	III	19	sustained VT	0.48
5	61/F	DCM	II	37	NSVT	0.43
6	52/M	myocarditis	II	42	VF	0.45
7	48/M	Tachycardia-induced cardiomyopathy	II	24	NSVT	0.48
8	64/F	Apical ballooning syndrome	II	47	none	0.43
9	61/F	myocarditis	II	42	none	0.48
10	45/M	DCM	II	50	none	0.39
11	55/M	DCM	III	40	sustained VT	0.51
12	37/M	DCM	II	40	none	0.47
13	55/M	DCM	II	38	none	0.47
14	42/M	DCM	II	20	none	0.46

NYHA: New York Heart Association Functional Classification, LVEF: left ventricular ejection fraction, DCM: idiopathic dilated cardiomyopathy, HCM: hypertrophic cardiomyopathy, VT: ventricular tachycardia, NSVT: non-sustained ventricular tachycardia, VF: ventricular fibrillation, QTc: corrected QT interval

More than half of the patients in the VT/VF (+) group took cardiotonic agents, whereas none of the patients in the VT/VF (−) group took cardiotonic agents (Table 2). However, none of the drugs, including cardiotonic agents, taken by the subjects have been reported to activate KCNH2 current.

**Table 2:** Patient's medication.

Case No.	Medications						
	Antiarrhythmics	Beta- blockers	Diuretics	ACEI/ARB	Anticoagulant/ Antiplatelet	Cardiotonic	others
1	amiodarone	carvedilol	torasemide	candesartan	none	none	none
2	none	carvedilol	furosemidespironolactone	valsartan	none	pimobendan	nateglinide, acarbose
3	none	carvedilol	furosemidespironolactone	imidapril	none	dopaminedobutaminemilrinone	lansoprazole
4	amiodarone	carvedilol	torasemide	losartan	warfarin	dopaminedobutamine	none
5	none	carvedilol	furosemide	candesartan	warfarin	none	alfacalcidol
6	sotalol	carvedilol	none	candesartan	warfarin	none	potassium chloride, famotidine
7	none	carvedilol	furosemidespironolactone	valsartan	warfarin	metildigoxin	lansoprazole
8	none	none	none	losartan	aspirin	none	nifedipine, famotidine, atorvastatin, icosapentate
9	none	carvedilol	furosemidespironolactone	losartan	warfarin	none	rabeprazole, ferrous citrate
10	none	none	none	none	none	none	none
11	none	carvedilol	torasemidetrichlormethiazide	candesartan	warfarin	metildigoxin	pravastatin
12	none	carvedilol	none	candesartan	none	none	amlodipine
13	none	carvedilol	furosemidespironolactone	candesartan	warfarinaspirin	none	none
14	none	carvedilol	furosemide	telmisartan	warfarin	none	amlodipine

ACEI/ARB: Angiotensin-Converting Enzyme Inhibitors/Angiotensin Receptor Blockers.

### Enhancement of KCNH2 currents by serum from HF patients with ventricular tachyarrhythmia

**Figure 1:**
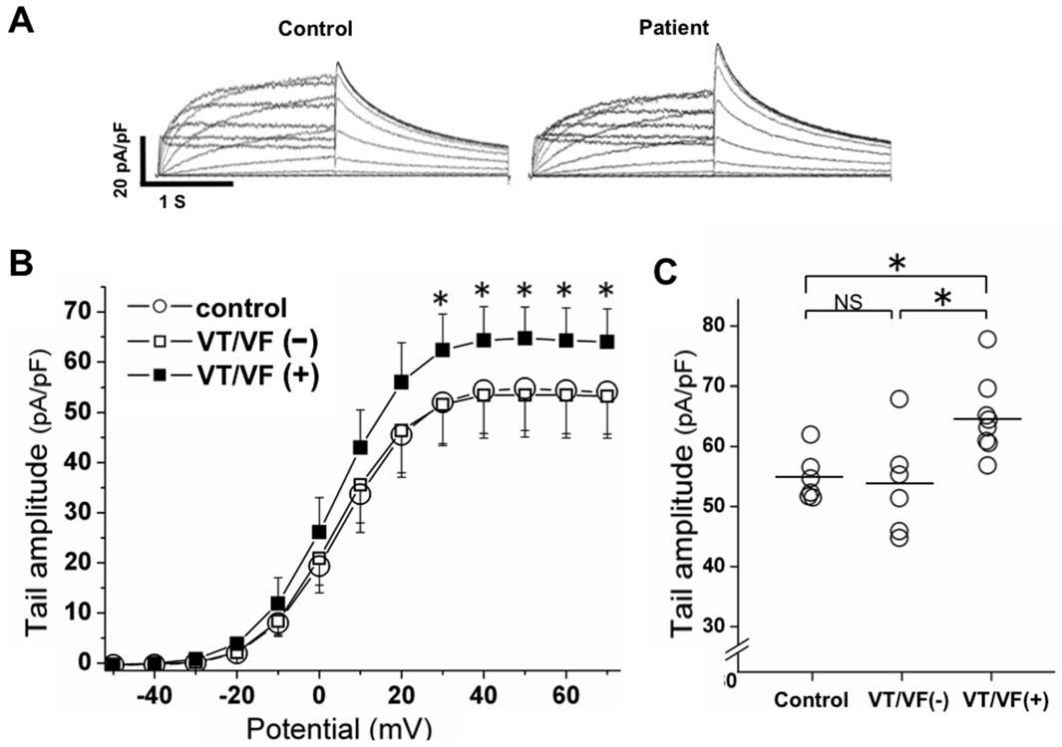
Effect of serum obtained from HF patients with ventricular tachyarrhythmia on KCNH2 currents in HEK 293 cells. A. Representative traces from a single cell cultured in a medium supplemented with 2% serum of a control subject (left) and medium supplemented with 2% serum of a patient with sustained VT (Case No. 1) (right). B. Current-voltage relationships of KCNH2 tail currents. Mean values of peak KCNH2 tail current densities were calculated by averaging the tail amplitude of each subject obtained from cells (n = 8 to 11 cells) exposed to respective sera. Open circles show the results for controls (n = 6), open squares show the results for HF patients without VT and VF (VT/VF (−), n = 6) and closed squares show the results for HF patients with VT/VF (VT/VF (+), n = 8). *P<0.05: VT/VF (+) vs. control and VT/VF (−). C. Maximum values of peak tail current in the groups. *P<0.05: VT/VF (+) vs. control and VT/VF (−). NS indicates not significant.

**Figure 2:**
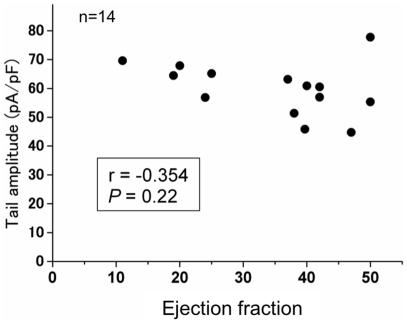
Relationship between peak tail amplitude and ejection fraction in the overall HF patients.

We investigated the effect of 2% serum of the study subjects on recombinant I_Kr_ recorded from HEK293 cells stably expressing KCNH2. KCNH2 tail currents were similar in the control and VT/VF (−) group but were significantly increased in the VT/VF (+) group (*P*<0.05: VT/VF (+) vs. control and VT/VF (−)) (Figure 1). There was no significant effect on voltage dependency of KCNH2 activation in these groups (Table 3). There was no relationship between peak tail amplitude and EF in the overall HF patients (Figure 2).

These results indicate the presence of a circulating KCNH2 channel activator in patients with HF and ventricular tachyarrhythmias.

**Table 3:** Changes in voltage dependence of the KCNH2 activation.

	Controls	VT/VF(−)	VT/VF(+)	*P*
V_1/2_ (mV)	5.6±4.8	3.7±3.5	4.3±2.5	NS
Slope factor (*k*)	8.2±0.8	8.2±0.9	8.6±1.0	NS

VT/VF (−): HF patients without VT and VF, VT/VF (+): HF patients with VT and VF NS indicates not significant.

### Efficacy of class III antiarrhythmic agents

Case No.1 was treated with amiodarone, and Case No.6 was treated with d-sotalol. Both drugs, which exert their antiarrhythmic actions essentially by blocking I_Kr_,were effective in controlling VT and VF.

## Discussion

The major new finding of this work is that the factor causing activation of the KCNH2 current is present in serum of patients with HF accompanied by cardiac arrhythmia. Although there are several reports on suppression of I_Kr_ by circulating hormones or auto-antibodies, this is the first report providing evidence for a naturally occurring I_Kr_ activator.[5], [11], [12] I_Kr_ conducted by KCNH2 channels is the major repolarizing outward current of ventricular action potential, and it has been shown to have a strong association with life-threatening arrhythmia in many pathological circumstances. Our finding indicates that enhancement of I_Kr_ can play a key role in arrhythmogenesis in the setting of HF.

The majority of research efforts have focused classically on reduction of I_Kr_ as an approach for understanding arrhtymogenesis because it is well recognized that reduction of I_kr_ means decrease in net repolarizing current that results in prolongation of action potential duration (APD) and QT interval in the ECG and development of early afterdepolarization-induced triggered activity following torsade de pointes (TdP).[13] On the other hand, much attention has been paid to upregulation of KCNH2 in recent years because of its causal relationship to cardiac arrhythmias. SQT is a recently recognized clinical concept characterized by short QT intervals in the ECG, and it is associated with major cardiac events leading to SCD without organic heart disease.[7] The first identified form of SQT resulted from gain-of-function mutation in KCNH2. Mutation N588K in KCNH2 causes an increase in net repolarizing current.[6] In addition, recent studies have revealed a significant association between early repolarization and SQT in clinical phenotype.[14], [15] Because of the high prevalence of early repolarization detected in SQT, it is thought that enhancement of repolarization is a common mechanism underlying arryhthmogenicity in early repolarization and SQT.[14] Since QT interval is affected by other ion channels and cardiac enlargement, QT interval of HF patients with ventricular tachyarrhythmia in this study was not short. However, the circulating KCNH2 channel activator in this study is potentially responsible for electrical instability.

Effects of KCNH2-activating agents have also been evaluated. It has been reported that PD-118057, established as a selective KCNH2 current enhancer without affecting activation, confers inducibility of both ventricular tachyarrhythmia and atrial fibrillation.[8], [16] The functional similarity between this agent and circulating KCNH2-activating factor in having no significant effect on the voltage dependence of activation suggests a causal relationship of this intrinsic enhancer with arrhythmogenesis in HF. In addition, this concept is endorsed by the efficacy of class III antiarrhythmic agents for treatment of ventricular tachyarrhythmia in the study subjects. Since the dominant action of class III antiarrhythmic agents is to lengthen the APD by a reduction of I_Kr_, it is thought that the therapeutic effect of the agents against arrhythmia in the study subjects is manifested by offsetting oversupplied KCNH2 currents.

On the other hand, there are only a few reports on the antiarrhythmic potential of an I_Kr_ activator in specialized pathologic settings. PD-118057 prevented the early after-depolarization induced by class III antiarrhythmic agents, and NS3623 decreased the frequency of bradycardia-induced extrasystoles *in vitro*.[17], [18] However, these data were obtained only in constrained experimental conditions and couldn't refer clinical benefit for arrhythmogenic disorders such as long QT syndrome.

The mechanism by which exposure of ion channels to this factor activates the KCNH2 current remains to be elucidated. It is also uncertain whether the KCNH2-activating effect is the cause or consequence of HF-induced remodeling of action potential. However, exertion of their functional effects may be involved in arrhythmogenesis in HF. Further studies are needed to clarify these points.

In conclusion, our data show that a novel KCNH2 activator exists in serum of HF patients with ventricular tachyarrhythmia and is potentially responsible for electrical instability.

## Methods

All of the studies were approved by the Ethics Committee of Okayama University Graduate School of Medicine, Dentistry, and Pharmaceutical Sciences, and written informed consent was obtained from all patients before the procedure. The investigation also conforms to the principles outlined in the Declaration of Helsinki.

### Study population

Serum samples were obtained from 14 HF patients referred to Okayama University Hospital, Okayama, Japan between December 2007 and December 2008 who consented to undergo evaluation of the pathogenesis of HF. HF was defined as the presence of current or previous symptoms of exercise intolerance and EF of 50% and less with no other cause of exercise intolerance. Exclusion criteria included coronary heart disease, primary valvular heart disease, severe systemic disease, or severe pulmonary disease. The mean age of the patients was 52±10 years, and 8 patients (57%) were male. Control sera were obtained from 6 subjects without cardiovascular disease or abnormal ECG findings. Mean age of the control subjects was 43±15 years, and 4 subjects (67%) were male.

### Patch-clamp recordings

KCNH2 channel currents were recorded at room temperature (22±1°C) by using the whole-cell patch-clamp technique as previously described.[11] The control bath solution contained (mM): 132 NaCl, 4.8 KCl, 1.2 MgCl_2_, 2 CaCl_2_, 5 glucose, 10 Hepes, pH 7.4. Pipettes (2–4 MΩ resistances) were filled with a pipette solution containing (mM): 110 potassium aspartate, 5 K2-ATP, 11 EGTA, 5 Hepes, 1 CaCl_2_, and 1 MgCl_2_, pH 7.3. Currents were normalized to cell capacitance to give the measure of current density. There was no difference in membrane capacitance between the groups (14.1±2.0 pF, 14.2±2.8 pF, and 14.7±1.6 pF for the control, VT/VF (−) and VT/VF (+) groups, respectively; *P* = 0.88). KCNH2 channel tail-current amplitude was monitored at 0.1 Hz by analysis of peak deactivating tail current recorded at −40 mV after 2-s depolarizing test pulses to +20 mV from a holding potential (*V*_H_) of −80 mV. Current zero level (no activation) was determined by applying 25-ms pulses to −40 mV preceding the test pulses. HEK293 cells stably expressing KCNH2 were cultured for 1 day in a medium to which 2% serum obtained from the study subjects had been added. The KCNH2 current activation was studied by analysis of deactivating tail currents recorded at −40 mV after a series of 2-s test pulses from −50 mV to +70 mV (10 mV increments; *V*_H_, −80 mV). Pulse frequency was 0.1 Hz. Peaks of tail currents were plotted as function of the KCNH2 activation. Activation curves were analyzed with a fit of each data to the Boltzmann equation, *I*/*I*_max_ = *G*/*G*_max_ = {1+exp[−(*V*_m_−*V*_1*/*2_)/*k*]}^−1^. *G*/*G*_max_ is normalized chord conductance at *V*_m_ to the maximum chord conductance. *V*_1*/*2_ is the potential where the conductance is half-maximally activated, and *k* is the slope factor.

### ECG measurements

Twelve-lead ECGs were recorded to measure the parameters of repolarization. The QT interval was measured from the start of the QRS complex to the end of the T wave, defined as the return to the iso-electric baseline. They were corrected to heart rate using Bazett's formula: QTc (QT/√RR).

### Data Analysis

All values are presented as means±.D. Statistical significance was assessed with ANOVA followed by post hoc Scheffe's method. A value of *P*<.05 was considered statistically significant. pCLAMP 9.2 software (Axon Instruments) was used to both acquire and analyze data for the patch-clamp experiments. Graphical analyses were carried out using Origin 7.0 J software (Microcal). Statistical analysis was performed with StatView 5.0 (Abacus Concepts, Inc., Berkeley, CA, U.S.A.).
